# Identification and validation a TGF-β-associated long non-coding RNA of head and neck squamous cell carcinoma by bioinformatics method

**DOI:** 10.1186/s12967-018-1418-6

**Published:** 2018-02-28

**Authors:** Teng Huang, Wei Huang, Hong Lu, Bi-yun Zhang, Jun Ma, Di Zhao, Yi-jun Wang, Da-hai Yu, Xia He

**Affiliations:** 1 Affiliated Hospital of Nanjing University of TCM, Nanjing, 210000 Jiangsu China; 2 Department of Radiation Oncology, Jiangsu Hospital of Traditional Chinese Medicine, 155 Han Zhong Road, Nanjing, 210000 Jiangsu China; 3 Jiangsu Collaborative Innovation Center of Traditional Chinese Medicine (TCM) Prevention and Treatment of Tumor, Nanjing, 210000 Jiangsu China; 4 Department of Radiation Oncology, Jiangsu Cancer Hospital, Nanjing Medical University Affiliated Cancer Hospital, Cancer Institute of Jiangsu Province, 42 Bai Zi Ting Road, Nanjing, 210000 Jiangsu China

**Keywords:** Head and neck squamous cell carcinoma, TGF-β, EPB41L4A-AS2, EMT

## Abstract

**Background:**

The role of transforming growth factorβ (TGF-β)-induced tumor progression in advanced malignancy is well established, but the involvement of long non-coding RNAs (lncRNAs) in TGF-β signaling remains unclear. This study aimed to identify TGF-β-associated lncRNAs in head and neck squamous cell carcinoma (HNSCC).

**Methods:**

Expression profiling of lncRNAs was obtained using Gene Expression Omnibus and The Cancer Genome Atlas. Real-time quantitative PCR was used to analyze the expression of EPB41L4A-AS2 in HNSCC cell line. We used bioinformatics resources (DAvID) to conduct Gene Ontology biological processes and KEGG pathways at the significant level. Wound healing assay, cell migration and invasion assays, were used to examine the effects of EPB41L4A-AS2 on tumor cell metastasis in vivo. Protein levels of EPB41L4A-AS2 targets were determined by western blot.

**Results:**

A novel TGF-β-associated lncRNA, EPB41L4A-AS2, was found downregulated by TGF-β and associated with invasion and metastasis. The relationship of EPB41L4A-AS2 with the clinicopathological features and prognosis of HNSCC patients was evaluated. Bioinformatic analyses revealed that EPB41L4A-AS2 may be involved in processes associated with the tumor-associated signaling pathway, especially the TGF-β signaling pathway. Furthermore, a TGF-β-induced epithelial-to-mesenchymal transition (EMT) model was established. Low EPB41L4A-AS2 expression was determined, and overexpression of this gene inhibited cell migration and invasion in the EMT model. Moreover, EPB41L4A-AS2 suppressed TGFBR1 expression.

**Conclusions:**

EPB41L4A-AS2 might serve as a negative regulator of TGF-β signaling and as an effective prognostic biomarker and important target in anti-metastasis therapies of HNSCC patients.

**Electronic supplementary material:**

The online version of this article (10.1186/s12967-018-1418-6) contains supplementary material, which is available to authorized users.

## Background

Long non-coding RNAs (lncRNAs) are a class of transcripts that are longer than 200 nucleotides without a protein-coding capacity [1]. Mounting evidence has recently indicated that lncRNAs are aberrantly expressed in different cancer types and are tightly associated with disease processes [2, 3]. A growing number of lncRNAs have been determined to play significant roles in invasion and metastasis in malignant tumor [4]. For example, HOTAIR promotes invasion in cervical cancer by targeting the Notch pathway, whereas HULC enhances the metastasis of hepatocellular carcinoma through the miR-200a-3p/ZEB1 signaling pathway [5, 6].

Transforming growth factorβ (TGF-β) is a vital cytokine factor in tumorigenesis and tumor progression [7, 8]. TGF-β promotes tumor progression by enhancing microenvironment modification, distant metastasis, and cell invasion partly through its capability to induce EMT [9, 10]. EMT is an important progression in the early events of cancer cell invasion and metastasis by endowing tumor cells with enhanced motility and invasion potentials [11]. The role of TGF-β-induced EMT in tumor cell dissemination has been well established. TGF-β signaling is mediated through SMAD and non-SMAD pathways to regulate EMT in tumor [12]. Recent studies have suggested that lncRNA, which serves as a mediator, participates in TGF-β-induced EMT [13]. lncRNA ATB, which is a mediator of TGF-β signaling, binds the miR-200 family and then the induced EMT in hepatocellular carcinoma [1]. TGF-β-induced upregulation of malat1 promotes bladder cancer metastasis through associating with SUZ12 [14]. However, the physiological significance of such interaction of the TGF-β–lncRNA axis requires further investigation.

Head and neck squamous cell carcinoma (HNSCC), the sixth leading cancer worldwide, is an aggressive cancer form with high metastasis and recurrence rates [15, 16]. Understanding the diverse etiology and molecular heterogeneity of HNSCC is crucial to overcome the barriers to the progress of prognostic tools and treatments. As members of a new class of regulatory molecules, lncRNAs have received increasing attention because of their roles in the invasion–metastasis cascade [17]. However, the specific roles of lncRNAs in mediating the metastatic role of TGF-β have not been extensively studied. In this study, we utilized gene expression microarray data in the GEO and mined next-generation sequencing and clinical data HNSCC patients from TCGA to fill this research gap. We specifically determined the effects of TGF-β on lncRNA expression and the correlation of this expression with cancer cell invasion and metastasis. We aimed to identify an lncRNA signature that participates in TGF-β signaling in the invasion–metastasis cascade of HNSCC.

## Methods

### GEO and TCGA gene expression datasets

HNSCC gene expression data and corresponding clinical data were obtained from the TCGA (https://tcga-data.nci.nih.gov and https://genome-cancer.ucsc.edu) and GEO databases (http://www.ncbi.nlm.nih.gov/geo/). To investigate lncRNA and TGF-β expression and between normal and cancer tissues, we downloaded RNASeq datasets for 43 tumor-adjacent normal pairs from TCGA and GSE59652 (N = 7) from the GEO database. Level date 3 were downloaded from the TCGA data and the expression profiling platforms is RNA-seqv2. To investigate the TGF-β-induced lncRNA changes in HNSCC, GSE54800 (lncRNA expression profile activated by TGF-β promotes the invasion–metastasis cascade in hepatocellular carcinoma, N = 14) was downloaded. To investigate the lncRNA signature related to invasion and metastasis in HNSCC, the data of 127 HNSCC patients were obtained from the TCGA database. These data consisted of 39 patients with and 88 patients without metastasis or recurrence. The exclusion criteria were set as follows: (i) histologic diagnosis is not HNSCC; (ii) suffering from other malignancies aside from HNSCC; and (iii) patients samples without complete data for analysis.

### RNA sequence data processing and computational analysis

**Fig. 1:**
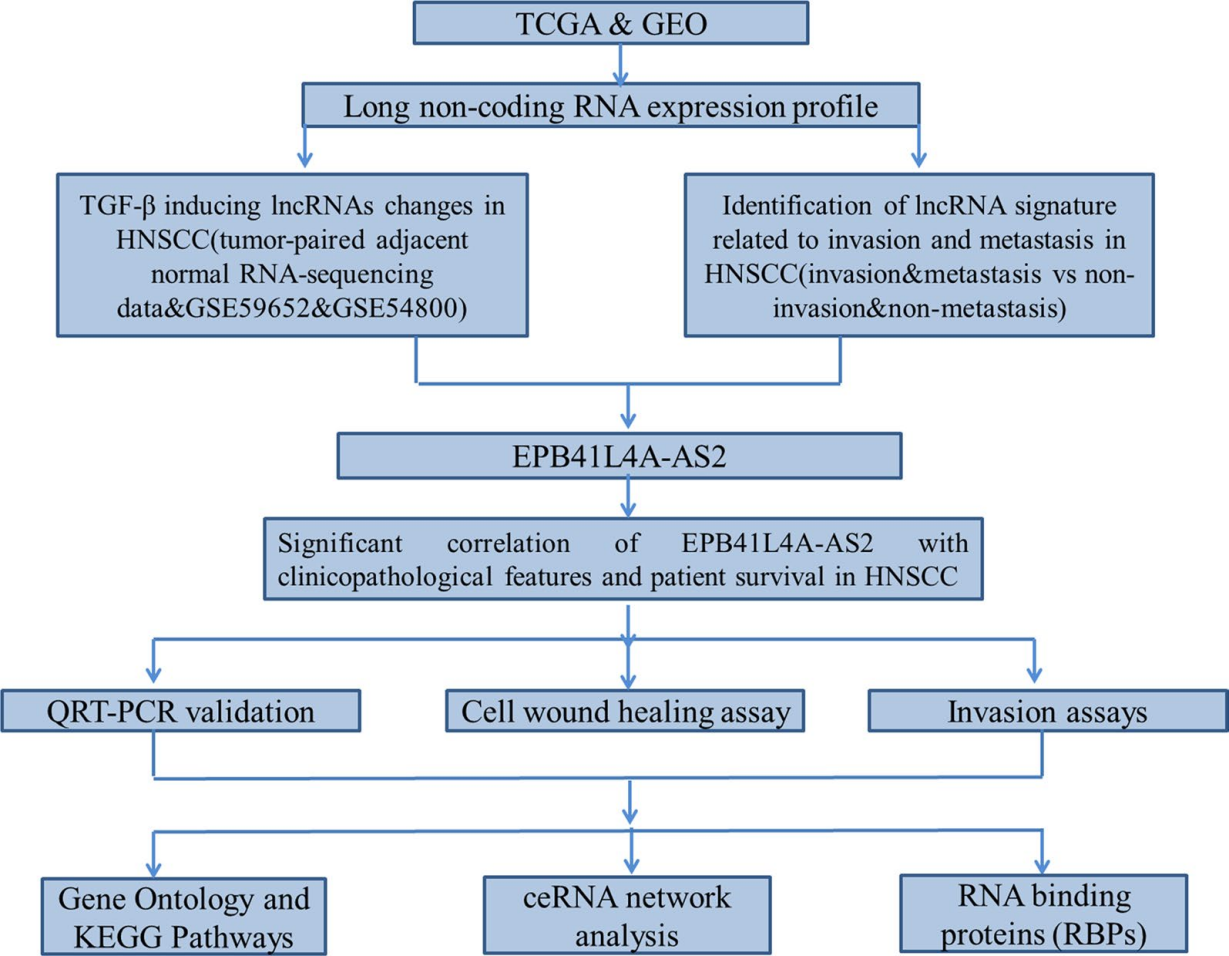
The flow chart for bioinformatics analysis

The RNA sequencing raw reads were post-processed and normalized by TCGA RNASeqV2 system. Level 3, normalized miRNA expression data were downloaded from the TCGA data portal performed using Illumina HiSeq and Illumina GA microRNA sequencing platforms and quantile normalized before performing analysis. No further normalizations were applied to the lncRNA, miRNA and mRNA expression profile data in level 3 [18, 19]. To detect the differential expression of lncRNA, miRNA, and mRNA, the samples were divided into different groups. For further analysis, the intersection of lncRNA, miRNA, and mRNA was selected. The flow chart for bioinformatics analysis is depicted in Fig. 1.

### Functional enrichment analysis

Functional enrichment analysis was actualized according to the literature [18, 19]. To understand further the underlying pathways and biological processes of aberrantly expressed lncRNAs, miRNAs, and mRNAs in HNSCC, we used bioinformatics resources (DAvID) (http://david.abcc.ncifcrf.gov/), to conduct Gene Ontology (GO) biological processes and KEGG (Kyoto Encyclopedia of Genes and Genomes) pathways at the significant level.

### Cell culture

NPC cell line CNE1 was obtained from the Research Center of Clinical Oncology of the Affiliated Jiangsu Cancer Hospital, Nanjing Medical University, Nanjing, China. CNE1 was propagated in RPMI 1640 medium (Hyclone, Logan, UT, USA) containing 10% FBS (Gibco BRL, Gaithersburg, MD, USA) and 1% antibiotic (Gibco BRL) and then incubated at 37 °C with CO_2_ saturation.

### Cell transfection

CNE1 cells were transfected with EPB41L4A-AS2 ORF clone (GeneCreate Biological Engineering Co., Ltd., Wuhan, China) in accordance with the manufacturer’s protocol. To determine the efficiency of transfection, the expression of EPB41L4A-AS2 was assessed with a qRT-PCR detection system (Bio-Rad, Hercules, CA, USA).

### Construction of EMT model

CNE1 cells were cultured in six-well plates (1 × 105 cells per well). Before coculture with TGF-β1 (10–15 ng/ml), the cells were cultured in RPMI 1640 medium without FBS for 24 h. Then, we treated CNE1 continuously with TGF-β1 (Peprotech, USA) for 7–10 days in RPMI 1640 medium containing 1% FBS.

### RNA sample preparation

Total RNA was extracted using TRIzol reagent (Invitrogen, Burlington, ON, Canada) in accordance with the manufacturer’s recommended protocol. The yield and purity of the RNA were determined by measuring the absorbance (Abs) at 260 and 280 nm. The RNA samples were only used when the Abs ratio of 260/280 nm was > 1.8.

### Quantitative reverse transcription-polymerase chain reaction analysis

RNA was purified using TRIzol reagent (Invitrogen, Carlsbad, CA) in accordance with the manufacturer’s instructions. Subsequently, 2 μg of total RNA was reverse-transcribed using Moloney murine leukemia virus reverse transcriptase (Promega, Madison, WI, USA) in accordance with the manufacturer’s instructions. The expression levels of lncRNAs were amplified using SYBR Green quantitative RT-PCR (qRT-PCR) on an ABI7300 real-time PCR machine (Applied Biosystems). The expression of EPB41L4A-AS2 and β-actin was examined using the following specific primers: EPB41L4A-AS2 forward primer 5′-GTCGCAGTTAGGGGAGACAC-3′ and EPB41L4A-AS2 reverse primer 5′-TGGCTACCCAGCTAACAAGC-3′ and β-actin forward primer 5′-GGACTTCGAGCAAGAGATGG-3′ and β-actin reverse primer 5′-AGCACTGTGTTGGCGTACAG-3′. Fold changes of lncRNA expression levels were calculated using the 2^−ΔΔCt^ method.

### Cell wound healing assay

CNE1 cells were cultured in six-well plates (2 × 105 cells per well) and then transfected with EPB41L4A-AS2 ORF clone. When the cells reached 75–90% confluence, a single wound was created by using a T-200-Y pipet tip. Wound areas were visualized under an optical microscope with a magnification of 100×. Cell migration capability was measured by gap closure.

### Migration and invasion assays

For migration assays, CNE1-transfected cells (5 × 104) were plated on the top chamber with a non-coated membrane. For invasion assays, CNE1-transfected cells (5 × 104) were plated on the top chamber with a coated membrane. In both assays, the cells were plated on the top chamber in medium without serum; the lower chamber was filled with 20% FBS. After 24–36 h of incubation, cells that invaded the membrane were fixed with 4% paraformaldehyde and then stained with crystal violet. Six random fields of cells were counted in each well under a microscope at a magnification of 100×.

### Construction of ceRNA network

Bioinformatic procedure of ceRNA network was actualized according to the literature [18, 19]. The lncRNA–miRNA interactions were predicted using miRanda tools (http://www.microrna.org/microrna/home). miRTarBase (http://mirtarbase.mbc.nctu.edu.tw/) and TargetScan (http://www.targetscan.org/) were used to predict the mRNAs targeted by miRNAs. The above results could be verified in the starBase v2.0 database (http://starbase.sysu.edu.cn/). Moreover, the predicted miRNAs and aberrantly expressed data of TCGA were combined to select the intersecting lncRNAs and mRNAs. Cytoscape v3.0 (30) was performed to construct and visualize the lncRNA–miRNA–mRNA ceRNA network.

### Statistical analysis

One-way ANOVA and Student’s t test were performed with SPSS 13.0 and GraphPad Prism 5.0 software. The significance level was set at 0.05 as default to control the false discovery rate. Data were expressed as the mean ± standard deviation of at least three separate experiments. Values of p < 0.05 were considered statistically significant.

## Results

### TGF-β upregulation in HNSCC and association with worse patient survival

**Fig. 2:**
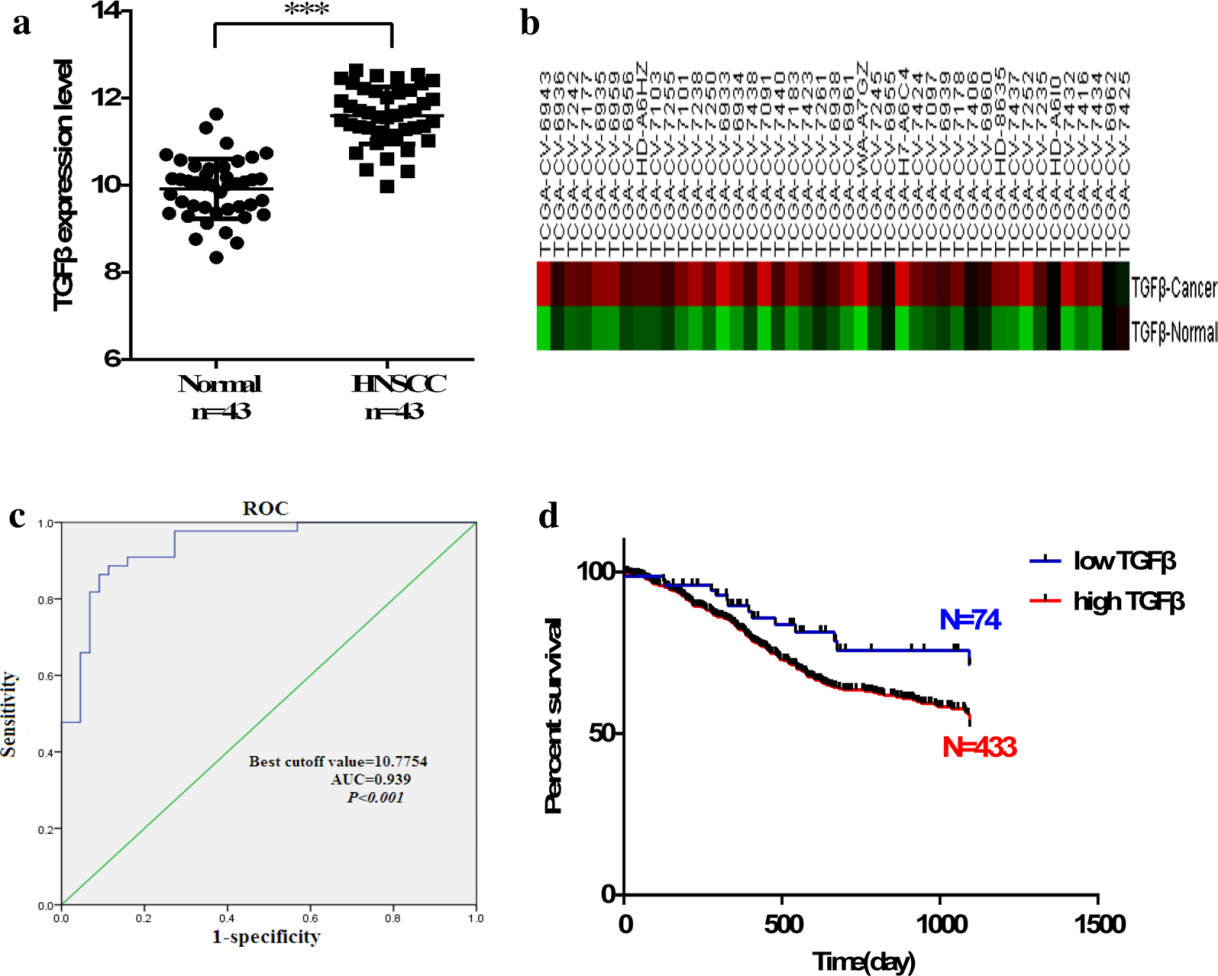
TGFβ upregulation in HNSCC and association with worse patient survival. **a**, **b** TGFβ is upregulated in HNSCC. **c** ROC analysis was performed to determine the cutoff value of TGFβ that could differentiate between the HNSCC patients with low and high TGFβ levels (p < 0.001; area under the ROC curve, 0.939; cutoff value, 10.775). **d** Based on the expression levels of TGFβ Kaplan–Meier survival analysis, patients with high TGFβ expression had significantly worse overall survival than those with low TGFβ expression. The log-rank test was used to calculate p value. *** p < 0.001

The expression levels and clinical significance of TGFβ were analyzed based on available samples in TCGA to determine the TGFβ expression profile in HNSCC and evaluate patient survival as a function of TGFβ expression. We downloaded RNAseq datasets for 43 tumor-adjacent normal pairs and determined that TGFβ expression was higher in the HNSCC samples than in the normal samples (Fig. 2a, b). The cutoff value of TGFβ was determined by receiver-operating characteristic analysis, which was employed to differentiate low and high TGFβ levels among patients (Fig. 2c). 507 HNSCC samples were downloaded from TCGA. On the basis of the expression levels of TGFβ, Kaplan–Meier survival analysis was performed. Results showed a negative association between high TGFβ expression and overall survival of HNSCC patients (Fig. 2d).

### Dysregulated TGF-β-associated lncRNAs in HNSCC

**Fig. 3:**
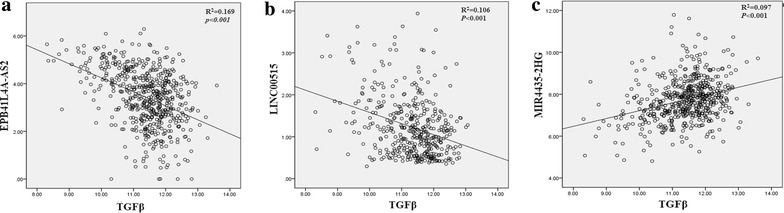
Three dysregulated TGF-β-associated lncRNAs were found in HNSCC. **a**, **b** A negative correlation was observed between EPB41L4A-AS2 as well as LINC00515 and TGF-β. **c** A positive correlation was observed between MIR4435-2HG and TGF-β

TGF-β-induced lncRNA profile in HNSCC is unavailable. Thus, we employed the TGF-β-induced lncRNA profile in hepatocellular carcinoma (GSE54800) as the lncRNA pool to identify the specific TGF-β-associated lncRNAs profile in HNSCC. Analysis of 50 tumor-adjacent normal pairs from TCGA and GEO (GSE59652) revealed 90 dysregulated lncRNAs in tumor tissues paired with normal tissues (Additional file 1). Many established and cancer-linked lncRNAs were observed in our results. Among the upregulated lncRNAs, Lnc00152 promotes proliferation in gastric cancer through the EGFR-dependent pathway [20]. HCP5 regulates the malignant behavior of glioma cells [21]. Among the downregulated lncRNAs, MEG3 inhibits lung cancer tumor progression through MYC downregulation [22]. Many lncRNAs with unexplored roles in HNSCC were revealed in our analysis, such as SFTA1P, FIRRE, and FKBP9P1. Considering the intersection GSE54800, our analysis identified three dysregulated TGF-β-associated lncRNAs in HNSCC (Table 1). EPB41L4A-AS2 and LINC00515 were downregulated, whereas MIR4435-2HG was upregulated. HNSCC studies associated with these lncRNAs are unavailable. Furthermore, we measured the expression levels of these three lncRNAs in HNSCC samples from TCGA. A negative correlation was observed between EPB41L4A-AS2 as well as LINC00515 and TGF-β (Fig. 3a, b). A positive correlation was observed between MIR4435-2HG and TGF-β (Fig. 3c).

**Table 1:** Three dysregulated TGFβ-associated lncRNAs in HNSCC

Gene ID	Symbol	lncRNA_Refseq	Fold-change TCGA	Style TCGA	Fold-change GSE54800	Style GSE54800	Fold-change GSE59652	Style (GSE59652)
54508	EPB41L4A-AS2	NR_027706.1	− 0.34	Down	− 4.61	Down	− 2.30	Down
282566	LINC00515	NR_024092.1	− 0.66	Down	− 3.01	Down	− 2.31	Down
541471	MIR4435-2HG	NR_136162.1	2.13	Up	2.47	Up	1.45	Up

### Identification of lncRNA signatures related to invasion and metastasis in HNSCC

We compared the levels of each lncRNA expression in the patient samples to identify the HNSCC-dysregulated lncRNA associated with invasion and metastasis. Considering that the clinical data of the patients were often incomplete, we focused on patients with the most complete set of criteria in terms of tumor status and follow-up data. We identified 167 dysregulated lncRNA transcripts (Table 2, Additional file 2). Our results confirmed several of the previously reported lncRNAs in HNSCC. HOTAIR, for instance, promotes tumor cell invasion and metastasis by recruiting EZH2 and repressing E-cadherin in oral squamous cell carcinoma [5]. Several lncRNAs (e.g., LINC00964, DICER1-AS1, and PPIEL) with unexplored roles in the invasion and metastasis of HNSCC were revealed in our analysis. Moreover, many other established invasion- and metastasis-linked lncRNAs showed differential expression. TUBA4B, MEG3, and SNHG12 were upregulated, whereas TUG1, OIP5-AS1, and LOC400927 were downregulated in HNSCC [23–28]. Finally, our results indicated that EPB41L4A-AS2 was downregulated not only in cancer tissues but also in metastasis or recurrence tissues.

**Table 2:** A part of lncRNA signatures related to invasion and metastasis in HNSCC

Gene ID	Symbol	lncRNA_Refseq	Fold-change TCGA	Style TCGA	*p* value
100132103	FAM66E	NR_027424.1	0.9	Down	0.027
152024	LINC00691	NR_026834.1	0.89	Down	0.046
90011	KIR3DX1	NR_026716.2	0.87	Down	0.032
145165	ST13P4	NR_002183.1	0.86	Down	0.031
285359	PDCL3P4	NR_002941.2	0.85	Down	0.018
119369	NUDT9P1	NR_002779.1	0.84	Down	0.048
286101	ZNF252P	NR_023392.1	0.83	Down	0.024
440465	BAIAP2-AS1	NR_026857.1	0.82	Down	0.044
728640	FAM133CP	NR_027508.1	0.82	Down	0.014
54508	EPB41L4A-AS2	NR_027706.1	0.71	Down	0.040
80086	TUBA4B	NR_003063.1	2.01	Up	0.0021
157381	LINC00964	NR_027321.1	1.96	Up	0.0014
55384	MEG3	NR_046473.1	1.87	Up	0.00717
400242	DICER1-AS1	NR_015415.1	1.77	Up	0.00024
100124700	HOTAIR	NR_003716.3	1.76	Up	0.016
728448	PPIEL	NR_003929.2	1.74	Up	0.002
286076	BREA2	NR_015445.1	1.69	Up	0.0012
100133036	FAM95B1	NR_026759.1	1.66	Up	0.017
11209	MST1P2	NR_027504.1	1.64	Up	0.028
284185	LINC00482	NR_038080.1	1.64	Up	0.038

### Significant correlation of EPB41L4A-AS2 with clinicopathological features and patient survival in HNSCC

**Fig. 4:**
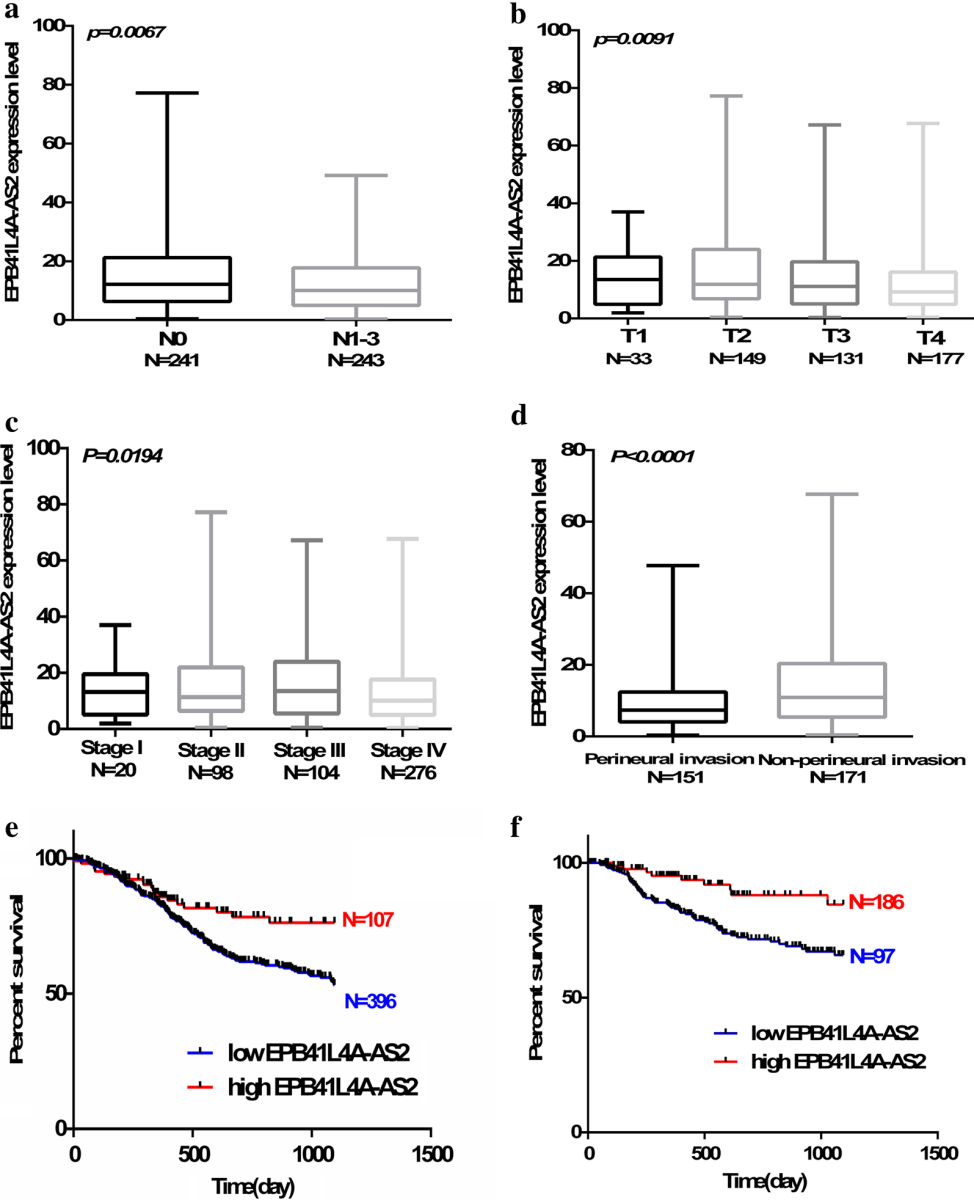
Significant correlation of EPB41L4A-AS2 with clinicopathological features and patient survival in HNSCC. **a**–**c** A negative relationship between EPB41L4A-AS2 expression and *N* classification, *T* classification, and tumor stage was found. **d** Low EPB41L4A-AS2 expression significantly correlated with patients with perineural invasion. **e**, **f** High EPB41L4A-AS2 expression was significantly positively associated with OS and RFS in HNSCC

Clinical characteristics for the total HNSCC cohort used are provided in Additional file 3. Low EPB41L4A-AS2 expression significantly correlated with increasing *N* classification, *T* classification, and tumor stage (Fig. 4a–c). Moreover, EPB41L4A-AS2 was aberrantly upregulated in HNSCC patients with perineural invasion (Fig. 4d). The relationship between EPB41L4A-AS2 expression and patient survival was analyzed. The cutoff value of EPB41L4A-AS2 was determined by receiver-operating characteristic analysis, which was employed to differentiate low and high TGF-β levels among patients (Additional file 4). High EPB41L4A-AS2 expression was significantly positively associated with a poor 3-year overall survival (OS) and relapse-free survival (RFS) in HNSCC (Fig. 4e, f). These data suggest that EPB41L4A-AS2 associated with disease progression and may has anti-oncogenic activity.

### EPB41L4A-AS2 is downregulated in the CNE1-EMT model

**Fig. 5:**
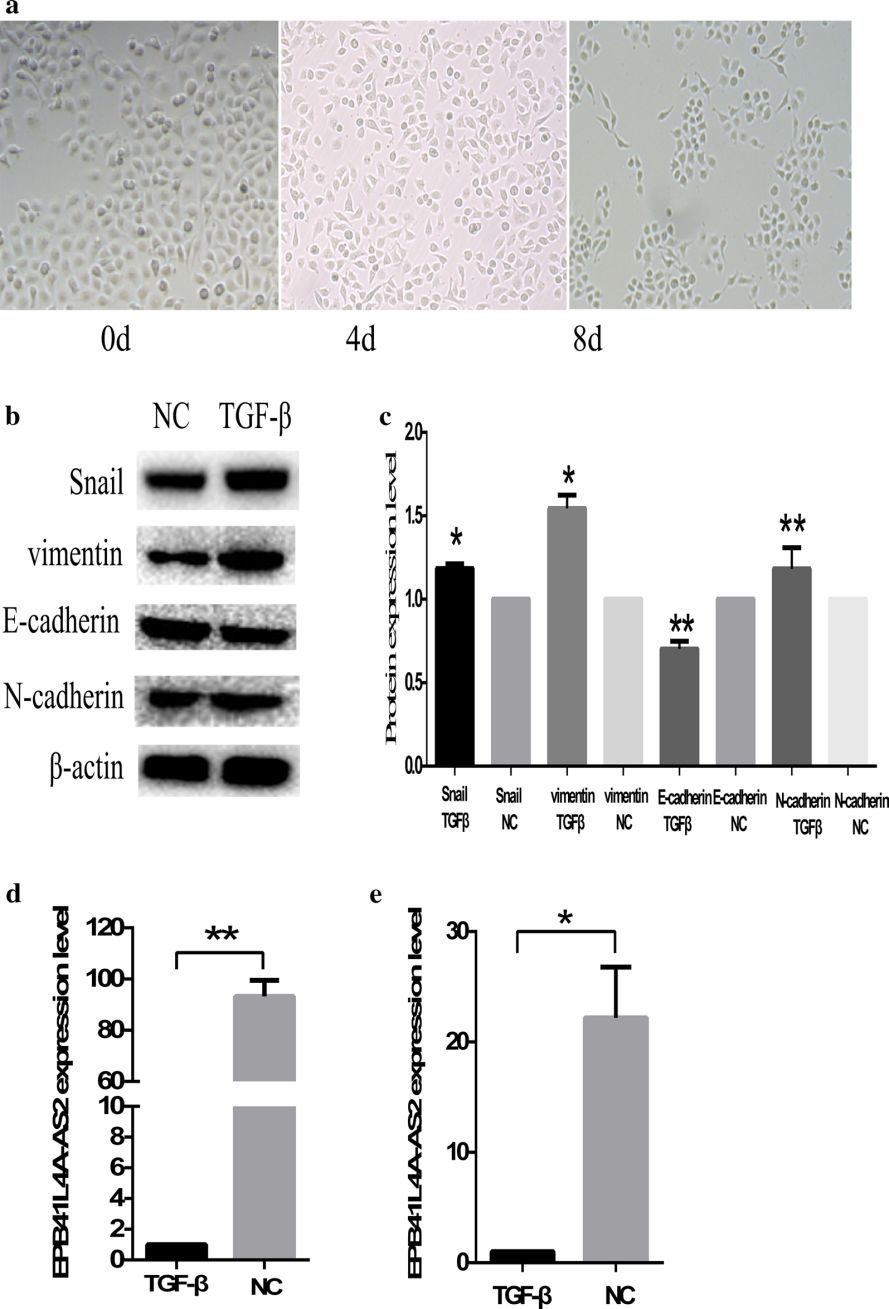
EPB41L4A-AS2 is downregulated in the CNE1-EMT model. **a** TGF-β-induced cell scattering and morphologic changes in CNE-1 cells. **b** Western blot analysis to detect the expression of Snail, vimentin, E-cadherin, N-cadherin and β-actin. **c** The relative protein expression levels were represented as columns. **d** The expression of EPB41L4A-AS2 was downregulated in TGF-β-induced EMT model. **e** EPB41L4A-AS2 expression level in the 3-day TGF-β treatment was downregulated. * p < 0.05 and ** p < 0.01

We selected the nasopharyngeal carcinoma line CNE1 to establish an EMT model in HNSCC for result verification [29]. CNE1 was treated continuously with TGF-β for 8 days, which caused the CNE1 cells to undergo EMT (Fig. 5a). CNE1 was indicated by a spindle-shaped appearance, increased Snail, vimentin and N-cadherin expression levels, and decreased E-cadherin expression (Fig. 5b, c). QPCR assays revealed that TGF-β induced a large decrease in EPB41L4A-AS2 expression in the CNE1-EMT model (Fig. 5d). Moreover, the EPB41L4A-AS2 expression level was also downregulated in the 3-day treatment (Fig. 5e).

### EPB41L4A-AS2 inhibits cell migration and invasion in the CNE1-EMT model

**Fig. 6:**
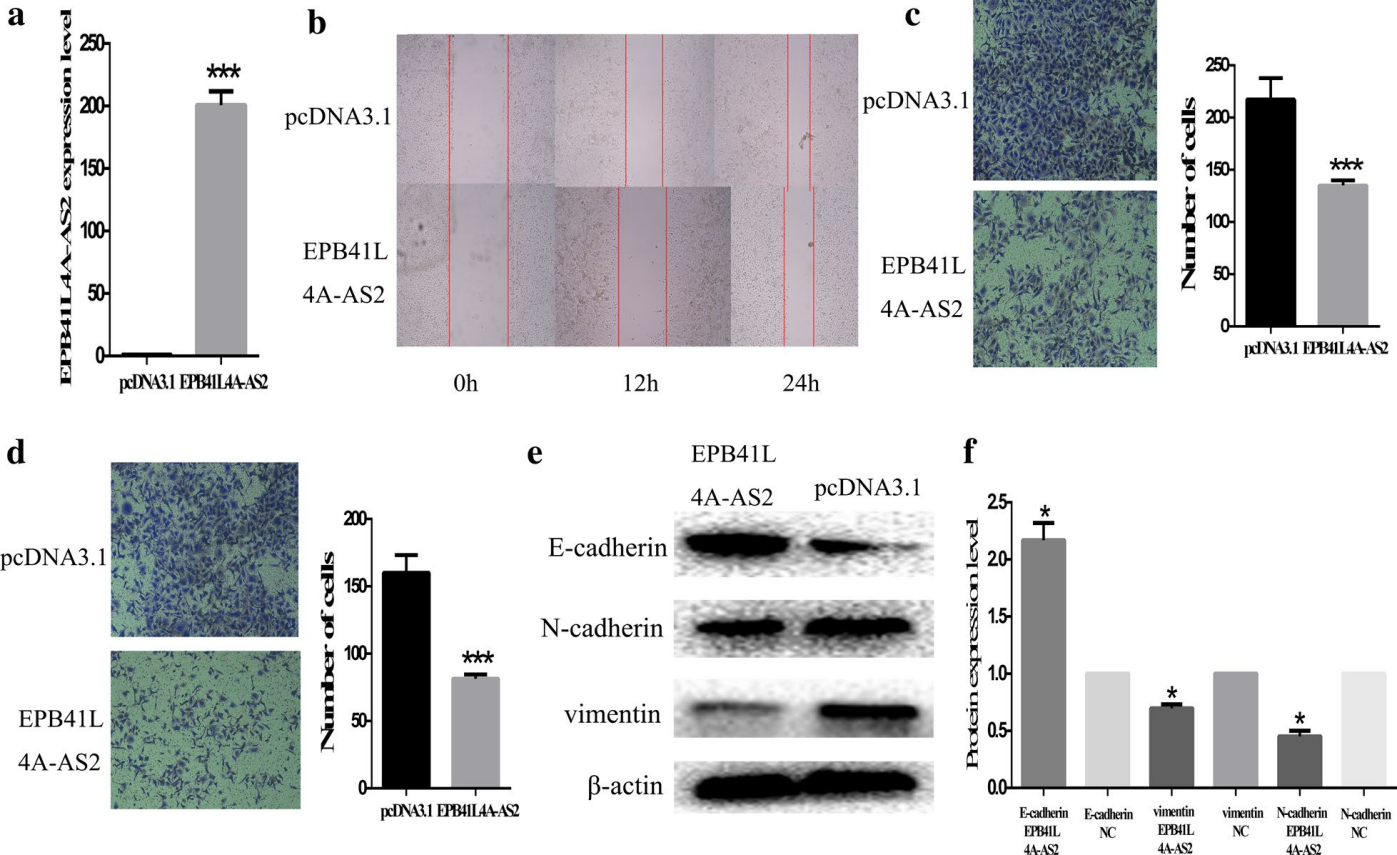
EPB41L4A-AS2 inhibits cell migration and invasion in the CNE1-EMT model. **a** Quantification of EPB41L4A-AS2 mRNA expression by qRT-PCR. **b** Effect on migration by wound healing assays. **c** Effect on migration by transwell assays. **d** Effect on invasion by transwell assays. **e** Western blot analysis to detect the expression of E-cadherin, vimentin, N-cadherin and β-actin. **f** The relative protein expression levels were represented as columns. * p < 0.05 and *** p < 0.001

TGF-β expression was upregulated in HNSCC as previously described. Therefore, we detected the function of EPB41L4A-AS2 in the EMT model to approximate the environment in vivo. CNE-1-EMT was transfected with pcDNA3.1-EPB41L4A-AS2. The successful overexpression of EPB41L4A-AS2 in CNE-1-EMT was confirmed by qPCR (Fig. 6a). The transfected CNE1-EMT was subjected to a wound-healing assay. Results showed that overexpression of EPB41L4A-AS2 decreased the migration capability of the CNE1-EMT model (Fig. 6b). Moreover, the transfected cells were cultured on a transwell apparatus. The numbers of migrated cells significantly decreased upon overexpression of EPB41L4A-AS2 (Fig. 6c). Furthermore, a matrigel model was established to examine the role of EPB41L4A-AS2 in invasion. The upregulation of EPB41L4A-AS2 decreased the invasion capability of the CNE1-EMT model (Fig. 6d). Moreover, overexpression of EPB41L4A-AS2 suppressed the expression of vimentin and promoted that of E-cadherin (Fig. 6e, f). These findings indicate that EPB41L4A-AS2 restrained cell migration and invasion in the CNE1-EMT model, as well as may participate in TGF-β-induced EMT.

### EPB41L4A-AS2 might inhibit the TGFBR1/SMAD2/SMAD3 axis

**Fig. 7:**
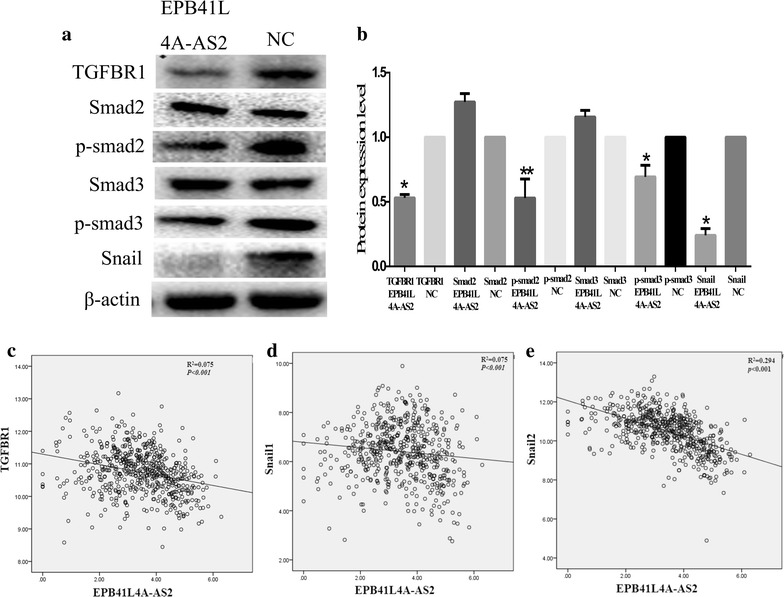
EPB41L4A-AS2 inhibits the TGFBR1/SMAD2/SMAD3 axis. **a** Western blot analysis to detect the expression of TGFBR1, Smad2, p-Smad2, Smad3, p-Smad3, Snail and β-actin. **b** The relative protein expression levels were represented as columns. **c**–**e** A negative correlation was found between EPB41L4A-AS2 and TGFBR1 or Snail1/2. * p < 0.05 and ** p < 0.01

The aforementioned evidence suggests that EPB41L4A-AS2 may be a negative regulator of the TGF-β pathway. Previous reports indicated that the TGFBR1/SMAD2/SMAD3/Snail axis plays a vital role in TGF-β-induced EMT [30]. We established that overexpression of EPB41L4A-AS2 suppressed TGFBR1 expression and inhibited SMAD2/SMAD3 phosphorylation and Snail expression (Fig. 7a, b). We also measured the expression levels of EPB41L4A-AS2 and TGFBR1 in 563 HNSCC tissues from TCGA. A negative correlation was found between EPB41L4A-AS2 and TGFBR1 or Snail1/2 (Fig. 7c–e). These findings suggest that EPB41L4A-AS2 may suppress TGF-β-induced EMT by inhibiting TGFBR1 expression.

### Analyses of the potential molecular mechanisms of EPB41L4A-AS2 by bioinformatics-based methods

The function of lncRNA involves a wide spectrum of biological contexts. The specific molecular mechanisms of EPB41L4A-AS2 have not been investigated in HNSCC. Our preliminary study indicated that EPB41L4A-AS2 inhibited TGFBR1/SMAD2/SMAD3 axis, but the specific mechanism by which EPB41L4A-AS2 participates in TGF-β signaling requires further investigation. Bioinformatics has recently become an effective approach to explore the potential molecular mechanisms of novel lncRNA. LncRNAs that target RNA-binding proteins (RBPs) are the vital mechanisms of lncRNA functions [4, 31]. Identifying the predicted target RBPs provides a basis for understanding lncRNA functions. Thus, the RBPs of EPB41L4A-AS2 were analyzed using the RBP prediction program RBP database (http://rbpdb.ccbr.utoronto.ca/). A part of analysis result are provided in Table 3. Among the different RBPs, several proteins have been proven to be associated with EMT. MBNL1 is a negative regulator of the TGF-β-dependent EMT of atrioventricular canal endocardial cells [32]. KHSRP silencing rewires post-transcriptional programs during TGF-β-induced EMT transition [33]. YBX1/YB-1 induces partial EMT and tumorigenicity through the secretion of angiogenic factors into the extracellular microenvironment [34].

**Table 3:** A part of analysis result of RBPs

Score	Relative score	RBP name	Start	End	Matching sequence
11.384	0.85	SFRS7	726	734	CCGAGAGAC
11.0709	1	A2BP1	498	503	UGCAUG
8.784	1	sap-49	39	44	GUGUGA
8.717	1	PABPC1	1136	1140	AAAAA
8.669	1	RBMY1A1	1130	1134	CUCAA
8.608	0.91	EIF4B	1270	1276	GUAGGAA
7.369	1	FUS	496	499	GGUG
7.229	1	Pum2	1269	1272	UGUA
7.0865	1	SFRS9	1260	1264	AGGAC
6.933	0.94	ACO1	861	866	CAGUGA
6.628	1	MBNL1	1142	1145	UGCU
6.340	1	KHSRP	826	829	GUCC
6.339	1	YBX1	351	356	CCUGCG
6.303	0.98	Vts1	563	569	GCUGGCC
6.236	1	YTHDC1	286	291	GAAUGC
5.268	1	RBMX	120	123	CCAG
5.0875	0.99	SFRS13A	1258	1264	AAAGGAC
4.841	1	RBM4	811	814	CGCG
4.620	1	SFRS1	1260	1263	AGGA
4.403	1	ELAVL1	1085	1088	GUUU
4.326	0.92	KHDRBS3	1373	1378	AAUAAA
11.384	0.85	SFRS7	726	734	CCGAGAGAC
11.0709	1	A2BP1	498	503	UGCAUG

**Fig. 8:**
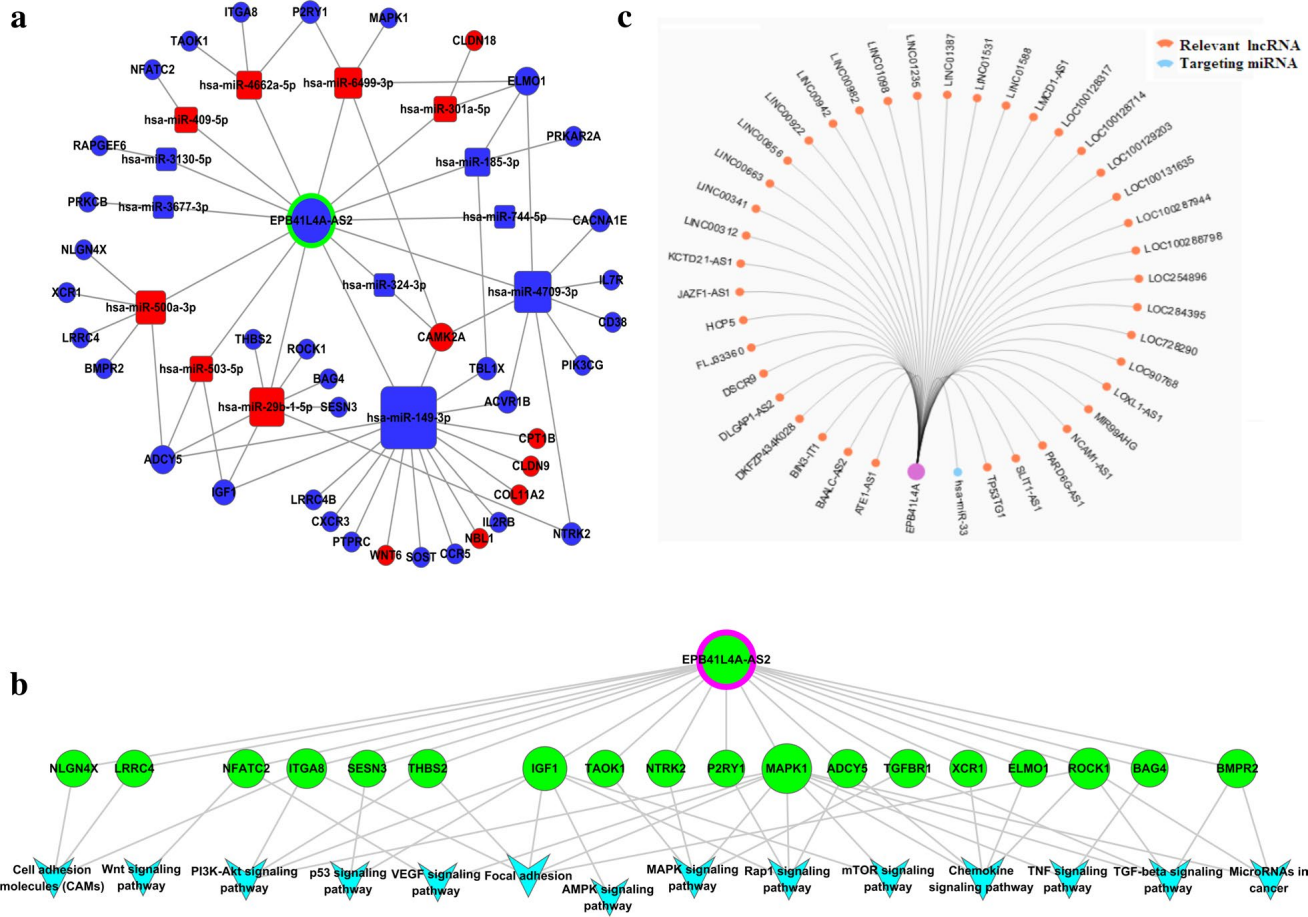
Analyses of the potential molecular mechanisms of EPB41L4A-AS2 by a bioinformatics-based method. **a**, **b** ceRNA and lncRNA-mRNA pathway network was established. **c** Regulatory network of EPB41L4A was constructed

Competing endogenous RNA (ceRNA) is currently under intense study as a novel mechanism for lncRNA functions, which indicates that RNA transcripts communicate with one another by miRNA response elements [31]. The ceRNA network of EPB41L4A-AS2 was constructed (Fig. 8a). Several miRNAs have contributed to EMT. For instance, miR-301a acts as an oncogene in laryngeal SCC and induces EMT in prostate cancer [35]. mRNAs from the ceRNA analysis serve as the target genes, and the lncRNA-mRNA pathway network was constructed (Fig. 8b). Several pathways are involved in the carcinogenesis of HNSCC metastases and invasion, such as the “PI3 K-Akt signaling pathway”, “Wnt signaling pathway”, and “mTOR signaling pathway”. Moreover, several pathways, such as the “MAPK signaling pathway”, “RAP1 signaling pathway”, and “AMPK signaling pathway”, are related to cancer development [36, 37].

Antisense transcripts have a wide variety of biological roles and play an important role in modulating transcription of sense RNA [38]. EPB41L4A-AS2 is the antisense RNA of EPB41L4A, which is a gene involved in the β-catenin/tcf signaling pathway [39]. We measured the expression levels of EPB41L4A-AS2 and EPB41L4A in 564 HNSCC samples from TCGA. A positive correlation was observed between EPB41L4A-AS2 and EPB41L4A (Additional file 5). Furthermore, we analyzed the regulatory network of EPB41L4A in GCBI (https://www.gcbi.com.cn/gclib/html/index) (Fig. 8c), so as to gain a better understanding of the mechanism of EPB41L4A-AS2.

## Discussion

In this study, we analyzed 50 tumor-adjacent normal pairs and identified 90 cancer-linked lncRNAs dysregulated in HNSCC relative to paired normal tissue. Then, TGF-β-induced lncRNA profile (GSE54800) was downloaded to identify the specific TGF-β-induced lncRNAs in HNSCC. Finally, our analysis confirmed three TGF-β-induced lncRNAs correlated with cancer. The key role of TGF-β-induced EMT is well established, and previous research has shown that EMT is a key molecular mechanism in tumor cell invasion and metastasis [40]. To identify the specific TGF-β-induced lncRNAs associated with invasion and metastasis in HNSCC, we compared the data of 39 patients with and 88 patients without distant metastasis or recurrence. Moreover, 167 lncRNAs were identified as differentially expressed. Finally, the expression of EPB41L4A-AS2 decreased in HNSCC and further decreased in distant metastasis or recurrence tissues. Moreover, a negative correlation was observed between EPB41L4A-AS2 and TGF-β in HNSCC tissues. Furthermore, we examined the relationship between EPB41L4A-AS2 expression and clinicopathological features in 507 patients with HNSCC. A lower level of EPB41L4A-AS2 was associated with T, N, and TNM stage in patients and was inversely correlated with prognosis. Therefore, EPB41L4A-AS2 was believed to have tumor suppressor gene activities and associated with the TGF-β signaling pathway.

Our results indicated that a low level of EPB41L4A-AS2 was associated with perineural invasion in patients with HNSCC, which is consistent with a previous finding that the biphasic activities of the TGF-β signaling pathway are also associated with perineural invasion [41]. Recent research has indicated that the level of TGF-β signaling pathway activity is higher in invasion and metastasis tissues than in primary HNSCC tissues, which further supports the regulation of EPB41L4A-AS2 by TGF-β in vivo [42].

Biphasic level of the TGF-β signaling pathway activity contributes to tumorigenesis, and recent research has indicated that TGF-β-induced lncRNAs inhibited the invasion–metastasis cascade of tumor in vivo [1]. Thus, the specific downstream lncRNAs of different TGF-β signaling pathways need to be explored further. In this study, the CNE1-EMT model was established by treatment with TGF-β. Our results indicated that EPB41L4A-AS2 was downregulated in the CNE1-EMT model, and overexpression of EPB41L4A-AS2 decreased the migration and invasion capability of the CNE1-EMT model. Moreover, overexpression of EPB41L4A-AS2 reversed the TGF-β-induced EMT. Thus, we identified that EPB41L4A-AS2 can be downregulated by TGF-β and mediates the role of TGF-β in inducing EMT. However, the mechanism by which TGF-β inhibits EPB41L4A-AS2 expression requires further investigation. Our results showed that a 3-day treatment with TGF-β was sufficient to downregulate EPB41L4A-AS2 expression, which implied that EPB41L4A-AS2 may be a direct target of the TGF-β/Smad pathway. Previous studies have identified that TGFBR1 is the vital receptor for TGF-β-induced EMT [30]. Our results indicated that overexpression of EPB41L4A-AS2 inhibited TGFBR1 expression. Furthermore, a negative correlation was found between EPB41L4A-AS2 and TGFBR1 in 563 HNSCC tissues from TCGA. However, the mechanism by which EPB41L4A-AS2 downregulates TGFBR1 expression requires further investigation. A major mechanism of lncRNAs in regulating gene expression involves the interaction with Polycomb repressive complex 2 (PRC2), which catalyzes the trimethylation of histone H3 lysine 27 (H3K27me3) to repress the transcription of specific genes [43]. Considering that the promoter region of TGFBR1 is rich in methylation, we hypothesized that EPB41L4A-AS2 plays a role in inducing the methylation of the TGFBR1 promoter (Additional file 6). The function of lncRNA involves many biological processes. We analyzed the specific molecular mechanisms of EPB41L4A-AS2 through a bioinformatics-based method. Our results indicated that EPB41L4A-AS2 may be a key participant in TGF-β signaling pathways through ceRNA or targeting RBPs. Several vital TGF-β signaling molecules, such as MBNL1 or miR-301a, may be regulated by EPB41L4A-AS2.

Overall, our research demonstrated that EPB41L4A-AS2 is downregulated by TGF-β and mediates the role of TGF-β in inducing EMT. EPB41L4A-AS2 inhibits TGFBR1 expression, which possibly regulates TGF-β signaling pathways. Our findings have significant implications regarding the understanding of TGF-β-lncRNAs in HNSCC. EPB41L4A-AS2 could be an effective target for anti-metastasis therapies.

## Conclusions

In summary, we revealed that EPB41L4A-AS2 is upregulated in HNSCC and downregulated by TGF-β. Our results indicated that EPB41L4A-AS2 might serve as a negative regulator of TGF-β signaling and as an effective prognostic biomarker. The newly identified EPB41L4A-AS2/TGFBR1/SMAD2/SMAD3 axis sheds light on a novel molecular mechanism for HNSCC cell metastasis, indicating that EPB41L4A-AS2 is a valuable biomarker and a promising therapeutic target for the management of HNSCC.

## Additional files

**Additional file 1.** Dysregulated lncRNAs in tumor tissues paired with normal tissues.

**Additional file 2.** Identification of lncRNA signatures related to invasion and metastasis in HNSCC.

**Additional file 3.** Demographics and clinical characteristics of TCGA HNSCC cohort.

**Additional file 4.** The cutoff value of EPB41L4A-AS2.

**Additional file 5.** A positive correlation was observed between EPB41L4A-AS2 and EPB41L4A.

**Additional file 6.** The methylation of the promoter region of TGFBR1.

## Abbreviations

HNSCC: head and neck squamous cell carcinoma
TCGA: The Cancer Genome Atlas
lncRNA: long non-coding RNA
EMT: epithelial-to-mesenchymal transition
NPC: nasopharyngeal carcinoma
ROC: receiver operating characteristic
AUC: area under the curve
NC: negative control
