# Supplementary material for: Identification and validation a TGF-β-associated long non-coding RNA of head and neck squamous cell carcinoma by bioinformatics method
Source: J Transl Med. 2018 Feb 28;16:46. doi: 10.1186/s12967-018-1418-6 (PMC5831574; doi:10.1186/s12967-018-1418-6)
Supplement: Supplementary file 3 — Additional file 3. Demographics and clinical characteristics of TCGA HNSCC cohort. [file 12967_2018_1418_MOESM3_ESM.docx]

| Gender | Male | 375 |
| --- | --- | --- |
|  | Female | 132 |
| Age | ≤ 61 | 269 |
|  | >61 | 238 |
| T classification | T1 | 33 |
|  | T2 | 149 |
|  | T3 | 131 |
|  | T4 | 177 |
|  | Not Available | 17 |
| N classification | N0 | 241 |
|  | N1-3 | 243 |
|  | Not Available | 23 |
| Clinical stage | I | 20 |
|  | II | 98 |
|  | III | 104 |
|  | IV | 276 |
|  | Not Available | 9 |
| Perineural Invasion | Yes | 151 |
|  | No | 171 |
|  | Not Available | 185 |

**Demographics and clinical characteristics of TCGA HNSCC cohort (*n* = 507)**
