# Supplementary figures and images for: Identification and validation a TGF-β-associated long non-coding RNA of head and neck squamous cell carcinoma by bioinformatics method
Source: J Transl Med. 2018 Feb 28;16:46. doi: 10.1186/s12967-018-1418-6 (PMC5831574; doi:10.1186/s12967-018-1418-6)

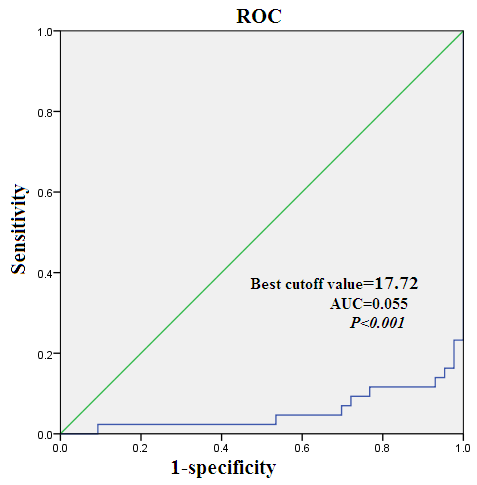

Supplement: Supplementary file 4 — Additional file 4. The cutoff value of EPB41L4A-AS2. [file 12967_2018_1418_MOESM4_ESM.tif]

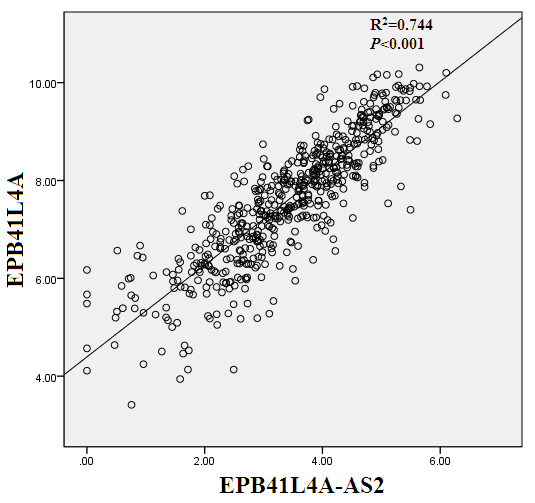

Supplement: Supplementary file 5 — Additional file 5. A positive correlation was observed between EPB41L4A-AS2 and EPB41L4A. [file 12967_2018_1418_MOESM5_ESM.tif]

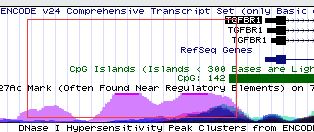

Supplement: Supplementary file 6 — Additional file 6. The methylation of the promoter region of TGFBR1. [file 12967_2018_1418_MOESM6_ESM.tif]
